# Gene expression patterns that support novel developmental stress buffering in embryos of the annual killifish *Austrofundulus limnaeus*

**DOI:** 10.1186/2041-9139-6-2

**Published:** 2015-01-21

**Authors:** Josiah T Wagner, Jason E Podrabsky

**Affiliations:** Department of Biology, Portland State University, P.O. Box 751, Portland, OR 97207 USA

**Keywords:** Annual killifish, Diapause, Gastrulation, Axis formation, Gene expression, Spemann-Mangold organizer

## Abstract

**Background:**

The cellular signaling mechanisms and morphogenic movements involved in axis formation and gastrulation are well conserved between vertebrates. In nearly all described fish, gastrulation and the initial patterning of the embryonic axis occur concurrently with epiboly. However, annual killifish may be an exception to this norm. Annual killifish inhabit ephemeral ponds in South America and Africa and permanent populations persist by the production of stress-tolerant eggs. Early development of annual killifish is unique among vertebrates because their embryonic blastomeres disperse randomly across the yolk during epiboly and reaggregate several days later to form the embryo proper. In addition, annual killifish are able to arrest embryonic development in one to three stages, known as diapause I, II, and III. Little is known about how the highly conserved developmental signaling mechanisms associated with early vertebrate development may have shifted in order to promote the annual killifish phenotype. One of the most well-characterized and conserved transcription factors, oct4 (Pou5f1), may have a role in maintaining pluripotency. In contrast, BMP-antagonists such as chordin, noggin, and follistatin, have been previously shown to establish dorsal-ventral asymmetry during axis formation. Transcription factors from the SOXB1 group, such as sox2 and sox3, likely work to induce neural specification. Here, we determine the temporal expression of these developmental factors during embryonic development in the annual killifish *Austrofundulus limnaeus* using quantitative PCR and compare these patterns to other vertebrates.

**Results:**

Partial transcript sequences to *oct4*, *sox2*, *sox3*, *chordin*, *noggin-1*, *noggin-2*, and *follistatin* were cloned, sequenced, and identified in *A. limnaeus*. We found *oct4*, *sox3*, *chordin*, and *noggin-1* transcripts to likely be maternally inherited. Expression of *sox2*, *follistatin*, and *noggin-2* transcripts were highest in stages following a visible embryonic axis.

**Conclusions:**

Our data suggest that embryonic cells acquire their germ layer identity following embryonic blastomere reaggregation in *A. limnaeus.* This process of cellular differentiation and axis formation may involve similar conserved signaling mechanisms to other vertebrates. We propose that the undifferentiated state is prolonged during blastomere dispersal, thus functioning as a developmental stress buffer prior to the establishment of embryonic asymmetry and positional identity among the embryonic cells.

**Electronic supplementary material:**

The online version of this article (doi:10.1186/2041-9139-6-2) contains supplementary material, which is available to authorized users.

## Background

The general course of early embryonic development is remarkably conserved between vertebrates, with developmental progression always following the same order: fertilization, cleavage, epiboly, gastrulation, axis formation, and organogenesis. Many of the morphogenic movements associated with these processes, such as gastrulation, are also conserved [1]. Barring developmental abnormalities or environmental insult, these stages typically progress in a unidirectional manner and without interruption. However, this is not the case in annual killifish development, which is characterized by a temporal separation of the morphogenic movements of epiboly from formation of the embryonic axis, and discontinuity due to naturally occurring periods of arrested development [2–4]. In this study we explore the temporal expression patterns of genes known to play key roles in the maintenance of pluripotency and the establishment of the vertebrate body plan during early development in embryos of the annual killifish *Austrofundulus limnaeus*.

*Austrofundulus limnaeus* (Order Cyprinodontiformes, Family Rivulidae) is an annual killifish found in ephemeral ponds of the Maracaibo Basin in northern Venezuela [5, 6]. Similar to other species of annual killifish, *A. limnaeus* maintains permanent populations by the production of drought- and anoxia-tolerant embryos [7–9] that are able to survive in the pond sediments after adult and juvenile fish are killed by habitat desiccation [3, 4, 10]. Tolerance of the environmental stresses imposed by their ephemeral environment is supported by the ability of the embryos to enter into a state of metabolic and developmental dormancy, termed diapause, at up to three distinct developmental stages [3, 4]. Diapause I (DI) may occur in some species of annual killifish during the dispersed blastomere stage prior to formation of an embryonic axis [11], although we do not regularly observe arrest at DI in our lab population of *A. limnaeus*. Diapause II (DII) can occur midway through development in an embryo that has undergone neurulation and segmentation, but is just prior to initiation of the major phases of organogenesis [4, 12]. Diapause III (DIII) can occur in the late pre-hatching embryo. Diapause II embryos display the highest resistance to abiotic stressors such as anoxia, salinity extremes, and desiccation when compared to other developmental stages [7, 8, 13].

In addition to the interruption of development by diapause, both the African and South American clades of annual killifish lack formation of a germ-ring or shield structure during epiboly [2, 3], which is atypical when compared to other described teleost fish species such as zebrafish (*Danio rerio*), the medaka (*Oryzias latipes*), and the mummichog (*Fundulus heteroclitus*) [14–16] as well as other non-annual killifish in the family Rivulidae such as *Kryptolebias marmoratus*[17]. Instead of the typical pattern of convergence and extension of the amoeboid (deep) embryonic blastomeres that is observed in most other teleost embryos during epiboly, deep blastomeres from annual killifish exhibit contact inhibition of cell movement [18, 19] and migrate away from each other across the yolk surface during epiboly where they remain dispersed across the yolk surface for several days [2]. These dispersed blastomeres later reaggregate, presumably through a delayed process of convergence and extension to form the definitive embryonic axis [2]. Although this dispersion and subsequent reaggregation process (D/R) was described several decades ago by Wourms [2], the molecular mechanisms that control these movements remains unexplored and the environmental and ecological relevance of this process have only recently been investigated [20, 21].

Underlying the gross morphological changes associated with embryogenesis are expression of inter- and intracellular signaling factors that encode for cellular identity and differentiation [22]. As developmental time progresses, embryos generally decrease expression of pluripotency genes in favor of genes that promote differentiation. In mammals, one of the most important factors required to maintain pluripotency *in vitro* is the co-expression of transcription factors oct4 (also known as Pou5f1) and a member of the SOXB1 family, sox2 [23, 24]. The transcription factor sox3, also a part of the SOXB1 family, likely precedes expression of sox2 during embryonic development and may have both unique and redundant functions with sox2 depending on the species studied [25–27]. Homologous genes to mammalian *oct4*, *sox2*, and *sox3* have been described in the zebrafish [28–30], and more recently in the medaka [31, 32]. Forming a complex with oct4, SOXB1 family transcription factors have a diverse array of targets during early fish development that are likely critical for normal developmental timing [33]. Whether annual killifish such as *A. limnaeus* express these pluripotency-promoting genes in a manner similar to other vertebrates is currently unknown. More importantly, the signaling mechanisms by which annual killifish embryos are able to transition from an undifferentiated blastula through a period of blastomere D/R prior to the formation of the embryo proper remain unexplored.

One of the most important periods of cellular differentiation in embryogenesis occurs during the process of axis formation, which follows the induction of gastrulation and establishes the organismal body plan. Diffusible signaling factors secreted by the Spemann-Mangold Organizer (SMO), a structure first described in 1924 [34], have an important role in establishing asymmetry in early vertebrate embryos [35]. In particular, correct dorsal-ventral (DV) patterning requires signaling gradients of bone morphogenic proteins (BMPs) and activin created by the expression of signaling antagonists by the SMO. The three major contributors to DV patterning through BMP inhibition are noggin, chordin, and follistatin. Chordin [36, 37] and noggin [38] are potent BMP antagonists, while follistatin [39, 40] antagonizes both BMPs and activin. Since the initial characterization of noggin, several noggins have been described [41, 42]. The requirement of noggin and chordin expression by the SMO to dorsalize embryos appears to be conserved between amphibians and fish, although follistatin appears be excluded from fish organizers [43–45].

It is currently unknown how blastomere D/R and the entrance into diapause II is regulated at the molecular level in *A. limnaeus embryos.* Additionally, the changes in expression of important developmental factors that are required to support the differences observed in early annual killifish development when compared to other teleosts are unclear. Although there are no morphological indications of embryonic patterning during epiboly in *A. limnaeus*, it has yet to be shown that cellular determination and differentiation does not occur during this period. Therefore, two major hypotheses for axis formation in annual killifish are: (1) cellular determination and differentiation occurs during epiboly, similar to other teleosts, and differentiated cells reaggregate later and segregate into germ layers to form an embryonic axis; or (2) cellular differentiation and therefore embryonic patterning does not occur until after reaggregation. The dynamic spatiotemporal expression patterns and cross-species conservation of *oct4*, *sox2*, *sox3*, *chordin*, *noggin*, and *follistatin* make these genes ideal candidates for characterization of pluripotency and axis formation in annual killifish.

Recently, we have reported that the D/R phases of development may act to buffer developing embryos from what would otherwise be teratogenic environmental insults [21]. The molecular mechanisms that support this unique buffering capacity remain to be resolved. If dispersed cells lack a unique cellular identity and location within the embryo, then cells lost or damaged during the D/R phase could presumably be replaced without negative consequences to the developmental program. This study describes for the first time the relative mRNA expression levels of genes critical for the maintenance of pluripotency and establishment of the embryonic axis across development in *A. limnaeus*, with the goal of comparing their patterns of expression to the highly conserved patterns noted in other vertebrates. The gene expression patterns reported here in *A. limnaeus* support a role for an extended period of pluripotency during the D/R phases of annual killifish development. This unique developmental pattern coupled with earlier reports of tolerance to cellular damage suggests that D/R can act as a buffering mechanism that supports normal embryonic development in the face of what would otherwise be teratogenic levels of cell damage and/or cell death due to environmental stress [21].

## Methods

### Husbandry of adults and treatment of embryos

Adult and embryonic *Austrofundulus limnaeus* were cared for as previously described by Podrabsky [46] and in accordance with approved Portland State University IACUC protocols. Mating pairs of fish were allowed access to spawning trays containing 1 to 2 cm of 500 μm glass beads (Thomas Scientific, Swedesboro, NJ, USA) for 2 h. Embryos were collected by sifting the glass beads through a 1.5 mm mesh and were transferred into embryo medium using a wide-mouthed plastic pipette. Fertilization was determined by the presence of a perivitelline space using a dissecting scope. Embryos were kept in embryo medium similar to the ionic composition of their native ponds (10 mmol 1^-1^ NaCl, 2.14 mmol 1^-1^ MgCl_2_, 0.8 mmol l^-1^ CaCl_2_, 0.14 mmol 1^-1^ KCl, 0.0013 mmol 1^-1^ MgSO_4_) with 0.0001% methylene blue added for the first 3 days post fertilization (dpf) to suppress fungal growth [5, 46]. At 4 dpf, embryos were treated with two 5 min washes of a 0.001% solution of sodium hypochlorite in embryo medium and transferred to embryo medium containing 10 mg l^-1^ gentamicin sulfate. Embryos sampled earlier than 4 dpf were treated with the bleaching regimen immediately before being flash-frozen as described below. Embryos were observed and embryo medium changed daily. Embryos were incubated at 25°C in darkness.

### Purification of total RNA from whole embryos and adult livers

#### Embryos

**Figure 1:**
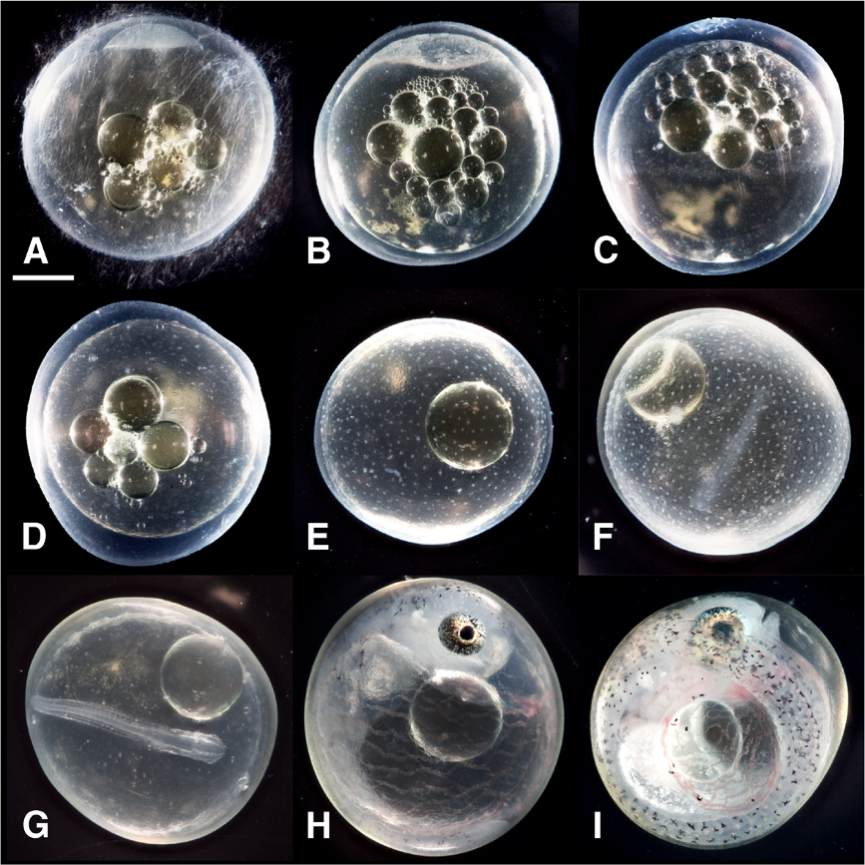
**Representative photographs of embryo stages used in this study. (A)** Early cleavage, **(B)** Early hollow blastula, **(C)** 50% epiboly, **(D)** Dispersed blastomere phases, **(E)** Reaggregation phases, **(F)** Solid neural keel, **(G)** Diapause II, **(H)** Three-quarter overgrowth, **(I)** Diapause III. Scale bar is 0.5 mm. Photos are from Riggs and Podrabsky, unpublished.

Embryos were observed using a dissecting microscope and sorted by stage as shown in Table 1 and Figure 1. Staged embryos were collected onto a nylon mesh screen (100 μm mesh), blotted dry with Kimwipes, transferred into 2 mL microcentrifuge tubes, and flash-frozen by submergence in liquid nitrogen. Embryos were stored at -80°C until RNA extraction.

**Table 1:** ***A. limnaeus*** **stages selected for qPCR analysis**

Stage	Age	WS ^a^	Abbreviation	Description	Pooled individuals	***N***
Early cleavage	3 hpf	3-5	EC	1-4 blastomeres	100	3
Early hollow blastula	1 dpf	12	EHB	Presence of a segmentation cavity containing blastomeres and covered by enveloping layer cells	100	4
50% epiboly	2 dpf	17	50% EP	Half of the yolk surface covered by periblast and enveloping layer. In between the two layers are embryonic blastomeres that have become ameboid and migrated away from the central blastula	100	4
Dispersed blastomere phases	4 dpf	20-21	DBP	Yolk surface completely covered by periblast, enveloping layer, and randomly distributed embryonic blastomeres	100	4
Reaggregation phases	8 dpf	22-26	RP	Embryonic blastomeres remain distributed across yolk, but a subpopulation are beginning to migrate towards a small area of the yolk where the future embryo will form. No discernable embryonic axis present	100	4
Solid neural keel	10 dpf	28	SNK	Presence of a solid neural keel, head fold, and Kupffer’s vesicle. No somites present	50	4
Diapause II	32 dpf	33	DII	Presence of optic cups and associated lenses, otic vesicles, functional heart, and 38 to 40 pairs of somites. Heart rate of 0 to 10 bpm	100	3
Three-quarter overgrowth	9 dpd	39	3/4 OG	Embryo occupies about three-fourths of the perimeter of the yolk. Eyes are heavily pigmented with gold colored material. Presence of incompletely developed gut, liver, and swimbladder	40	4
Diapause III	24 dpd	43	DIII	Fully formed larva that has completed embryonic development, but has not yet hatched	40	4
Adult liver	Adult	N/A	Liver	Whole liver from an adult female	1	3

^a^WS, Wourms’ Stage, stages based on [3].

dpd, Days post diapause; dpf, Days post fertilization; hpf, Hours post fertilization.

#### Adult livers

Adult *A. limnaeus* females were euthanized by immersion in ice water for several minutes followed by cervical transection. Livers were removed, transferred into 2 mL microcentrifuge tubes, flash-frozen in liquid nitrogen, and stored at -80°C until RNA extraction.

#### RNA extraction

Frozen embryos were immersed in TRIzol reagent (Invitrogen #15596-026) at a ratio of 50 μL TRIzol reagent per embryo. Frozen adult livers were immersed in TRIzol reagent at a ratio of 100 mg liver per mL of TRIzol. Immediately after addition of TRIzol, embryos or livers were homogenized using an IKA Ultra-Turrax T8 (Wilmington, NC, USA) at room temperature until lysis was complete. Homogenates were subjected to centrifugation at 10,000 × *g* for 30 min at 4°C to remove cellular debris. Supernatants were transferred into new tubes and 0.2 mL of chloroform per mL of TRIzol was added. Samples were gently mixed by vortexing and subjected to centrifugation for 20 min at 10,000 × *g* at 4°C. The clear aqueous phase was removed carefully and transferred into a fresh tube. RNA was precipitated using a high salt method by addition of equal volumes (1.25 mL of each per mL of TRIzol used) of a solution containing 0.8 M sodium citrate and 1.2 M NaCl and 100% isopropanol. Samples were vortexed gently and incubated at -20°C overnight to precipitate the RNA. The following day, samples were subjected to centrifugation at 10,000 × *g* for 30 min at 4°C. Supernatants were decanted carefully so as to not disturb the RNA pellet. The RNA pellet was washed with 1 mL of 60% ethanol (EtOH) followed by centrifugation at 10,000 × *g* for 30 min at 4°C. Supernatants were decanted and the wash step repeated. After the final wash, EtOH was removed and the pellet was centrifuged at 10,000 × *g* for 1 min at 4°C to collect residual EtOH. The residual EtOH was removed and remaining EtOH was allowed to evaporate for 10 to 15 min. RNA pellets were resuspended in 25 to 55 μL of 1 mM sodium citrate (pH 6.4). Incubation of samples for 4 to 5 min at 55°C facilitated RNA pellet resuspension. Sample concentrations and A_260_/A_280_ ratios were determined using the Infinite M200 Pro plate reader equipped with a NanoQuant plate (Tecan, San Jose, CA, USA) using 2 μL of sample and default software settings (i-control software, Tecan). RNA integrity was determined by agarose gel electrophoresis of 0.5 μg of total RNA and observing distinct banding for 18S and 28S rRNA subunits. Average A_260_/A_280_ ratios of 1.7 to 2.2 were routinely obtained with the exception of DII embryos, which had low ratios (mean 1.3 ± 0.12 SD) but retained 18S and 28S rRNA banding after gel electrophoresis that was comparable to other stages. Samples were stored at -80°C or used immediately as template for reverse transcription reactions.

#### Identification and PCR amplification of *A. limnaeus*RNA transcripts of interest

*Austrofundulus limnaeus* sequences for genes of interest were amplified from total RNA by polymerase chain reaction (PCR). Total RNA was reverse transcribed using the RevertAid first strand cDNA synthesis kit (Fermentas #K1621). Prior to addition of enzymes, RNA was mixed with primer, heated to 65°C for 5 min, and chilled rapidly on ice. Reverse transcription (RT) reactions (20 μL total volume) contained RNA (250 to 500 ng), 5 μM anchored oligonucleotide dT primer (sequence: 5′ TTT TTT TTT TTT TTT TTT TTV N 3′), 1 mM dNTP mix, 20 U RiboLock RNAse inhibitor, 200 U of RevertAid M-MuLV reverse transcriptase, in 1X RevertAid reaction buffer (50 mM Tris–HCl pH 8.3, 50 mM KCl, 4 mM MgCl_2_, 10 mM DTT). Reactions were incubated at 42°C for 60 min and were terminated by incubation at 70°C for 5 min. The single-stranded cDNA was used immediately for PCR or was stored at -20°C.

Prior to PCR amplification, remaining RNA from the RT reaction was degraded by incubation at 65°C for 15 min in 200 mM NaOH and 100 mM EDTA. Following RNA degradation, pH was neutralized by addition of 1 M Tris (pH = 7.5) to a final concentration of 20 mM. The ssDNA samples were purified using the QIAquick PCR purification kit (Qiagen #28104) according to the manufacturer’s instructions and were eluted in 30 μL of nuclease-free dH_2_O. RNA transcript sequences for genes of interest from other vertebrates (Additional file 1: Table S1) were identified using NCBI GenBank database searches. Degenerate or specific primers were used depending on sequence conservation between species and were created using PrimaClade online software [47]. The PCR parameters were adjusted according to specific primer pairs, and often one or more parameter had to be adjusted in order to cleanly amplify a particular gene of interest. In general, 1 to 5 μL of purified cDNA, 5 to 10 pmol of both forward and reverse gene-specific primer (Integrated DNA Technologies), 0.125 U *Taq* polymerase (New England BioLabs #M0267L), and 2.5 μL 10X ThermoPol buffer (New England BioLabs # M0267L) were used per 25 μL reaction. Reactions were cycled for 37 to 46 cycles with varying melting, annealing, and extension temperatures (Additional file 1: Table S1).

### Cloning and sequencing of genes

Creation of plasmids with cDNA fragments and growth of transformed bacterial cell cultures were performed based on the methods of Sambrook *et al.*[48]. DNA templates generated during PCR were analyzed by gel electrophoresis through a 1.5% agarose gel in 0.5X TBE for 30 to 45 min at 100 V. Fragment sizes were estimated by comparison to a GeneRuler 1 kb Plus DNA ladder (Thermo Scientific #SM1332). DNA fragments that were in the range of expected PCR product size were excised with a razor and purified using the QIAquick MinElute gel extraction kit (Qiagen #28606) according to manufacturer’s instructions. DNA was eluted in 10 μL of nuclease-free water and stored at -20°C or used immediately for cloning. Purified PCR products were cloned into the pGEM-T Easy Vector System (Promega #A1360). For each 10 μL cloning reaction, 50 ng of pGEM-T Easy Vector was mixed with 3 μL of purified PCR product and 3 Weiss units of T4 DNA ligase in 1X rapid ligation buffer. Samples were mixed and incubated overnight at 4°C. The next day, 2 μL of each ligation reaction was added to 20 to 30 μL of competent *Escherichia coli* cells (Strain JM109, Promega #L2001, >10^8^ cfu μg^-1^) in 1.5 mL microcentrifuge tubes. Samples were mixed gently and incubated on ice for 20 min. Cells were transformed by heat shock for 50 s in a 42°C water bath and were immediately returned to ice for 2 min. SOC medium (2% Bacto-tryptone, 0.5% yeast extract, 10 mM NaCl, 0.5 mM KCl, 10 mM MgCl_2_, 10 mM, MgSO_4_, 20 mM glucose) was added to each tube of cells (19 μL SOC medium per 1 μL of cells) and cells were transferred to sterile 15 mL polypropylene round-bottom culture tubes. Cells were incubated for 1.5 h at 37°C while shaking (150 rpm). Following incubation, 40 to 60 μL of the SOC cultures were plated onto agar screening plates (1.5% Bacto-agar, 1% Bacto-tryptone, 42.8 mM NaCl, 33.5 mM KCl, 1 mM CaCl_2_ 0.02 mg mL^-1^ X-Gal, 0.014 mg mL^-1^ IPTG, 0.1 mg mL^-1^ ampicillin sodium salt). Plates were incubated overnight at 37°C. White colonies were selected using a sterile toothpick and used to inoculate 1 mL of lysogeny broth (1% Bacto-tryptone, 0.5% yeast extract, 171 mM NaCl 0.1 mg mL^-1^ ampicillin sodium salt. Liquid cultures were incubated overnight at 37°C while shaking (200 rpm). The following day, cultures were subjected to centrifugation at 7,000 × *g* for 3 min at room temperature to pellet cells. Plasmids were purified from the cell pellet using the QIAprep Spin Miniprep Kit (Qiagen, #27104) according to manufacturer’s instructions and were eluted in 30 μL of nuclease-free water. Plasmid quantity was determined by measuring sample absorbance at 260 nm and quality was determined by the observation of A_260_/A_280_ ratios between 1.8 and 2.0. Purified plasmids were diluted with nuclease-free water and 500 ng of plasmid template was mixed with 6.4 pmol of pUC/M13 reverse primer (sequence 5′-TCA CAC AGG AAA CAG CTA TGA C-3′) in final volumes of 20 μL. Plasmids were submitted for Sanger sequencing at the Oregon Health and Science University DNA Services Core (Portland, OR, USA) using an Applied Biosystems 3730xl capillary sequencer. Sequenced plasmids were visualized using FinchTV software (Geospiza, v. 1.4, 2013) and vector sequences were removed to reveal cloned *A. limnaeus* sequences. Sequence identity was inferred using NCBI blastx (non-redundant protein database) or blastn (nucleotide database) searches for the seven genes of interest (Additional file 2: Table S2 and Additional file 3: Table S3). Partial mRNA sequences for *sox3* and *noggin-2* were identified during clone screening using primers for *sox2* and *noggin-1*, respectively. Sequences for *β-actin* and 18S rRNA were also cloned while screening for other genes.

### Reverse transcription of total RNA for quantitative PCR

Treatment of RNA and cDNA prior to quantitative PCR (qPCR) was based on previously described methods [49]. RNA samples were treated with DNAse enzyme to degrade possible genomic DNA contaminants. DNAse reactions consisted of 5.5 μg of total RNA for each sample, 2 U of RNAse-free DNAse I (New England Bio Labs #M0303S), 40 U of RiboLock RNase inhibitor (Thermo Scientific #EO0381), and 2 mM MgCl_2_, in a final volume of 16.5 μL. Samples were incubated at 37°C for 10 min followed by incubation at 90°C for 5 min. DNAse-treated total RNA (5 μg) was reverse transcribed into single-stranded cDNA using 1 μL of iScript advanced reverse transcriptase in 1X iScript advanced reaction mix (Bio-Rad #170-8842) in a final volume of 20 μL. The iScript advanced buffer contains both oligo(dT) and random primers. Samples were incubated at 42°C for 30 min followed by reaction termination at 85°C for 5 min. Samples were diluted 1:4 in nuclease-free water and stored at -20°C until use in qPCR.

### qPCR primer-probe design and reaction conditions

Primer and probe sequences to be used for qPCR were created using the PrimerQuest tool and purchased from Integrated DNA Technologies (Additional file 4: Table S4) [50]. For probe chemistry we used PrimeTime ZEN Double-Quenched Probes. Probes were 5′ labeled with fluorescein (FAM), internally labeled with a ZEN quencher, and 3′ labeled with an IBFQ quencher [51]. Probes are hydrolyzed by the 5′ > 3′ exonuclease activity of the DNA polymerase, freeing the quenchers from the FAM dye. The difference in estimated melting temperature (T_m_) between forward and reverse primers was designed to be no more than 5°C. Probes were selected to have melting temperatures approximately 5°C greater than the forward and reverse primers. Observation of a single amplicon of the appropriate estimated size on a 1% agarose gel following PCR amplification from single-stranded cDNA was used to verify primer specificity. The DNA sequence of each amplicon from each gene/primer set was verified by cloning and DNA sequencing as described above. Synthetic DNA standards identical to the amplicons generated by the qPCR primers were purchased from IDT (Additional file 2: Table S2). qPCR reactions were set up in triplicate using SsoFast Probes Supermix (Bio-Rad, #172-5230) and consisted of 1 μL of diluted cDNA, 10 μL of 2X SsoFast Probes Supermix, 500 nM of forward and reverse primers, and 250 nM of probe in final volumes of 20 μL. All reactions used this 1:2 ratio of primers:probe except for the oct4 assay which used a 1:1 ratio. Assays were set up in clear 96-well semi-skirted PCR plates (Hard-Shell High-Profile PCR plates, Bio-Rad, #HSS-9601) with optical flat caps (Bio-Rad, #TCS-0803). All qPCR reactions were carried out in a Stratagene Mx3005P thermocycler (Agilent Technologies, Santa Clara, CA, USA). Standard curves were generated using 1 × 10^-3^, 1 × 10^-4^, 1 × 10^-5^, and 1 × 10^-6^ copies of synthetic standard. Reactions were initially heated at 95°C for 30 s to activate the DNA polymerase and subsequently thermocycled for 40 cycles by denaturation at 95°C for 30 s and annealing/elongation at 60°C for 30 s. Fluorescence readings (excitation 492 nm, emission 516 nm) were taken at the end of each elongation step. Quantification cycle (C_q_) thresholds were set automatically in Stratagene MxPro software (ver. 4.10, 2007) using adaptive baseline, moving average, and amplification-based threshold settings. Although rarely necessary, thresholds were manually adjusted to improve standard curve best-fit regressions.

### Statistics

Fold changes for the genes were calculated relative to one of the SNK (10 dpf) samples using the efficiency corrected ddC_q_ method [52]. To correct for between-plate variation, we used the efficiency of the standards run on each plate for the fold-change calculations. As suggested by Bustin and Nolan (2004), C_q_ values within five cycles of the no template control or the 40th cycle were dropped from analysis to reduce the possibility of false positives [53]. Prior to statistical analysis, relative expression values were normalized relative to *β-actin* or 18S rRNA expression and log_2_ transformed. Differences in relative expression between developmental stages were calculated using one-way Analysis of Variance (ANOVA) followed by Tukey’s multiple comparison test. Statistical significance was determined at *P* <0.05.

## Results and discussion

### Identification of *A. limnaeus*transcripts

Partial mRNA sequences for *A. limnaeus oct4*, *chordin*, *sox2*, *sox3*, *noggin-1*, *noggin-2*, and *follistatin* were identified using the blastx sequence alignment tool against sequences in NCBI nucleotide databases for all vertebrates or against only *D. rerio* (Additional file 3: Table S3 and Additional file 5: Table S5). Putative conserved domains were also identified by sequence similarity using blastx. The *oct4* transcript fragment (278 bp) isolated from *A. limnaeus* included a POU-superfamily domain from nucleotide residues 1 to 75 (E = 1.52 × 10^-3^) and a DNA binding domain from nucleotide residues 136 to 276 (E = 1.13 × 10^-4^). The *A. limnaeus sox2* transcript fragment (287 bp) included a SOX transcription factor domain from nucleotide residues 35 to 286 (E = 1.94 × 10^-8^). The transcript fragment for *A. limnaeus sox3* (295 bp) included a SOX transcription factor domain from nucleotide residues 53 to 124 (E = 3.02 × 10^-3^) and a SOX-TCF HMG-box, class I domain from nucleotide residues 191 to 295 (E = 1.52 × 10^-6^). The isolated *A. limnaeus chordin* transcript fragment (773 bp) included a CHRD (chordin) superfamily domain from nucleotide residues 1 to 294 (E = 8.57 × 10^-9^) and a von Willebrand factor type C domain from nucleotide residues 403 to 537 (E = 1.91 × 10^-5^). The identified *A. limnaeus noggin-1* fragment included a Noggin superfamily domain from nucleotide residues 1 to 483 (E = 1.71 × 10^-66^) while the *noggin-2* fragment included a Noggin superfamily domain from nucleotide residues 1 to 243 (E = 1.72 × 10^-35^). The *A. limnaeus follistatin* transcript fragment included a Follistatin-like SPARC (secreted protein, acidic, and rich in cysteines) domain from nucleotide residues 71 to 286 (E = 2.31 × 10^-9^) and between 521 to 661 (E = 1.20e-09). The *follistatin* transcript also included a Kazal type serine protease inhibitor domain from nucleotide residues 176 to 280 (E = 3.19 × 10^-7^) and 362 to 505 (1.2 × 10^-9^). Nucleotide residues from 41 to 598 were identified as being a part of the high cysteine membrane protein group 4 (E = 7.82 × 10^-3^). For all genes tested, we observed dynamic expression profiles across development. Additionally, we observed high r^2^ values for the synthetic standards following qPCR (>0.99). We did not observe substantial differences in expression after normalization to either *β-actin* or 18S rRNA (not shown), and therefore we present data and focus our discussions on data normalized to expression of β-actin mRNA. Expression of *oct4*, *sox3*, *sox2*, *chordin*, *noggin-1*, *noggin-2*, and *follistatin* was not observed in adult liver samples, and therefore we focus our discussion on embryonic patterns of expression.

### Expression of pluripotency and neural differentiation regulators in *A. limnaeus*

#### oct4

**Figure 2:**
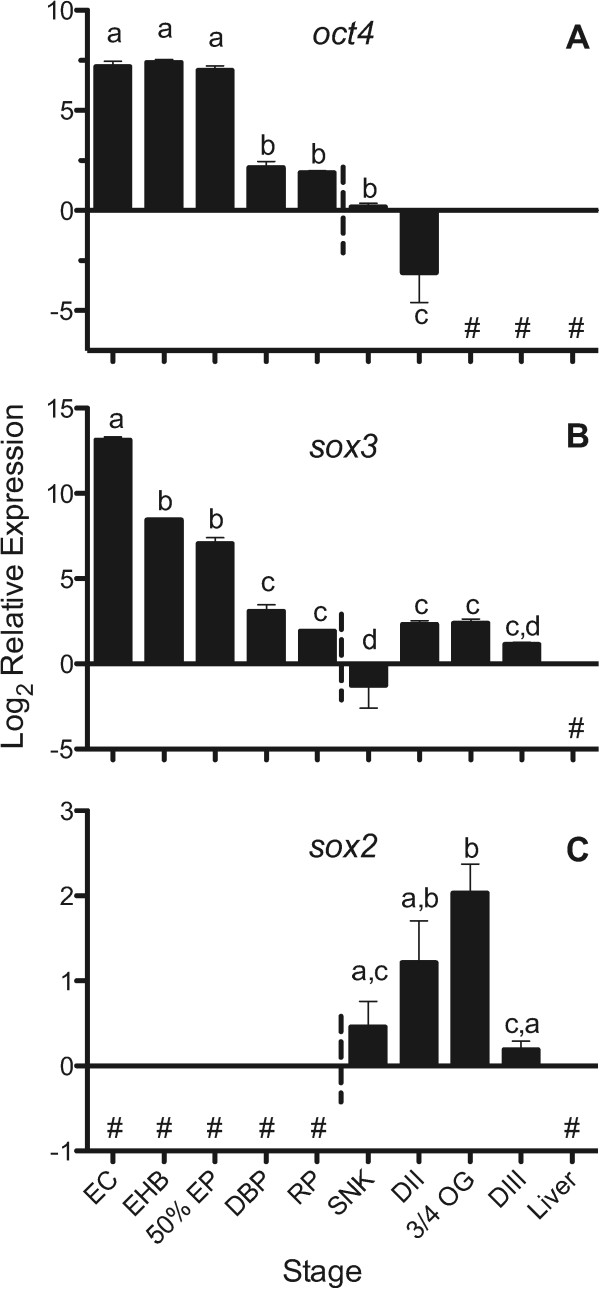
**Expression of putative** ***oct4*** **(A),** ***sox3*** **(B), and** ***sox2*** **(C) transcripts during** ***A. limnaeus*** **development.** Developmental stages to the left of the horizontal dashed line do not have a visible embryonic axis. Bars with different letters are statistically different (*P* <0.05, one-way ANOVA with Tukey’s post-test). Values are means ± SEM (*N* = 3–4). Stages with undetected expression are noted with #. EC, early cleavage; EHB, early hollow blastula; 50% EP, 50% epiboly; DBP, dispersed blastomere phases; RP, reaggregation phases; SNK, solid neural keel; DII, diapause II; 3/4 OG, three-quarters overgrowth; DIII, diapause III.

**Figure 3:**
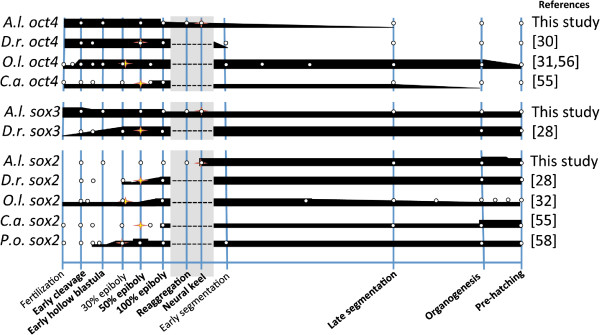
**Gene expression patterns of** ***oct4*** **,** ***sox3*** **, and** ***sox2*** **during development in** ***A. limnaeus*** **and other teleost fishes.** All data points (white dots) are inferred from available qPCR data. Thickness of the black bars represents relative expression levels within each species. The stage with the first visible evidence of embryogenesis is indicated with a star for all species. Developmental stages that are absent in species other than *A. limnaeus* are indicated with a gray box. For data from this study, late segmentation and organogenesis stages would correspond to the DII and 9 dpd stages, respectively. Similar developmental stages were categorized based on previously reported characterizations [14, 15, 58, 59]. *D.r*, *Danio rerio*; *O.l.*, *Oryzias latipes*; *C.a.*, *Carassius auratus*; *P.o.*, *Paralichthys olivaceus*.

The transcription factor oct4 is widely conserved between vertebrates and is often associated with its ability to maintain pluripotency during development. Homologs to mammalian Oct4/Pou5f1 have been described in a wide range of species, including *Xenopus*, zebrafish, medaka, the goldfish *Carassius auratus*, and the chicken [30, 31, 54]. Although the zebrafish *oct4* gene was initially known as *pou2*, the similarity to mammalian Oct4/Pou5f1 suggests that they are indeed orthologs, and thus the zebrafish *pou2* is considered to be equivalent to *oct4* in other vertebrates [33, 55]. Teleost expression of *oct4* mRNA begins very early during embryonic development [30, 31, 56, 57], suggesting maternal packaging, and similarly we found highest expression of *oct4* mRNA before the completion of epiboly in *A. limnaeus* embryos (Figure 2A). Expression of *A. limnaeus oct4* mRNA decreased over developmental time until becoming undetectable after DII. This pattern of *oct4* transcript expression (Figure 3) is similar to that found in zebrafish by Takeda *et al.*[30], in medaka by Wang *et al.*[31], and in goldfish by Marandel *et al.*[56], suggesting a conserved role for *oct4* in early development for *A. limnaeus*. Interestingly, whereas medaka oct4 may have similar functions to mammalian Oct4 in maintaining pluripotency, zebrafish oct4 may not be necessary for this purpose [54, 57]. Localization of *oct4* transcripts and protein in *A. limnaeus* embryos during development will be necessary to determine if the spatial expression of the transcription factor is similar to other described taxa.

#### sox2 and sox3

Similar to *oct4* transcripts, we detected a high abundance of *sox3* transcripts beginning in early *A. limnaeus* development (Figure 2B), which suggests it is maternally inherited. *sox3* transcript abundance was high during early cleavage, but expression generally decreased as development progressed (Figure 1B). In zebrafish, *sox3* mRNA expression is detected starting at the 32-cell stage (Figure 3; the earliest stage sampled), suggesting maternal inheritance, and is detectable until 48 hpf (early hatching) [28, 60]. We also observed *sox3* expression for the entire duration of *A. limnaeus* embryonic development, but in contrast to zebrafish, we observed the highest expression of *sox3* just following fertilization with a significant decrease in expression during early development leading to lowest expression at the SNK stage (10 dpf). *A. limnaeus sox2* transcripts were not detectable prior to reaching the SNK stage and had highest expression between DII and mid-organogenesis 3/4 OG (Figure 2C). In zebrafish, *sox2* expression is not observed until 30% epiboly, and is associated with initiation of gastrulation [28]. Similarly, *sox2* expression is not observed prior to epiboly in goldfish, with the first transcripts being detected at 75% epiboly [56]. Expression of *sox2* in *Xenopus* has been suggested to be activated by *sox3*, and thus it is not surprising we observed *sox3* expression prior to *sox2*[26]. In contrast to zebrafish and *A. limnaeus*, medaka *sox2* expression appears to be more transient, although the strongest expression of *sox2* is between the early neurula (1 dpf) and 16 to 19 somites (2 dpf) stages [32]. The Japanese flounder *Paralichthys olivaceus* also differs slightly from both zebrafish and medaka in that low expression of *sox2* mRNA is first observed in the high blastula and peak expression occurs in the early to mid-gastrula [61]. Although the expression patterns of *sox2* appear to differ across development in these teleosts, they share the pattern of increased expression near the beginning of gastrulation (Figure 3).

SOXB1 genes, including *sox2* and *sox3*, work with *oct4* to regulate neural fate and differentiation [62–64]. Expression of *oct4* early in development, and simultaneous expression of *sox2* and *sox3* genes at the SNK stage (presumably the time for neural induction) in *A. limnaeus* suggests that the function of these genes in regulating pluripotency and neurulation are conserved with other vertebrates. Interference with oct4 or sox3 function produces gastrulation defects in *Xenopus* and zebrafish [55, 65], and thus the expression of *oct4* and *sox3* in *A. limnaeus* embryos prior to axis formation also suggests a possible conserved role in gastrulation. In contrast to the strong upregulation of *sox3* at 30% epiboly in zebrafish [27], *sox3* is downregulated after 50% epiboly in *A. limnaeus*. The developmental consequences of this reversal in *sox3* expression during *A. limnaeus* development are unknown. Future studies that establish the molecular targets and localization of *oct4*, *sox3*, and *sox2* in *A. limnaeus* will be important in determining if the roles of these transcription factors are indeed similar to other vertebrates.

### Expression of DV-patterning genes during *A. limnaeus*embryonic development

Chordin, noggin, and follistatin are potent BMP antagonists that are commonly associated with their role in DV patterning. Gradients of BMP across the developing embryo are established by secretion of these BMP antagonists by the Spemann-Mangold organizer, a structure that forms during gastrulation and whose function in inducing dorsal structures appears to be conserved between vertebrates [35].

#### chordin

**Figure 4:**
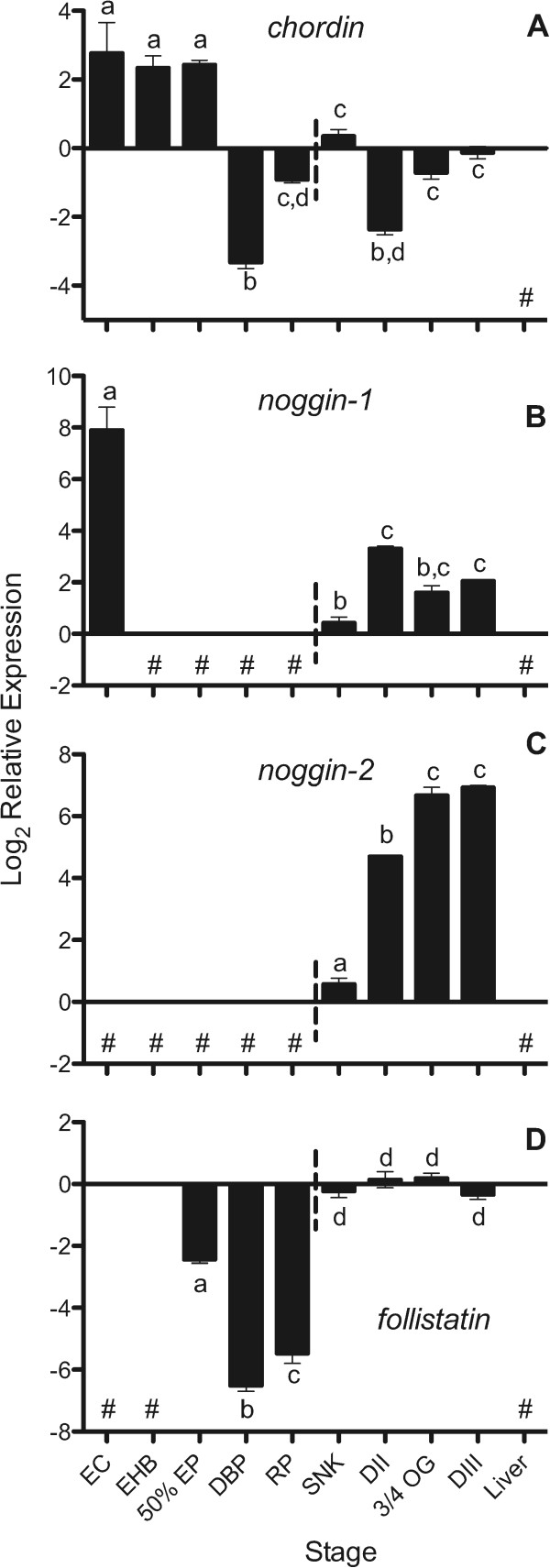
**Expression of putative** ***chordin*** **(A),** ***noggin-1*** **(B),** ***noggin-2*** **(C), and** ***follistatin*** **(D) transcripts during** ***A. limnaeus*** **development.** Developmental stages to the left of the horizontal dashed line do not have a visible embryonic axis. Bars with different letters are statistically different (*P* <0.05, one-way ANOVA with Tukey’s post-test). Values are means ± SEM (*N* = 3–4). Stages with undetected expression are noted with #. EC, early cleavage; EHB, early hollow blastula; 50% EP, 50% epiboly; DBP, dispersed blastomere phases; RP, reaggregation phases; SNK, solid neural keel; DII, diapause II; 3/4 OG, three-quarters overgrowth; DIII, diapause III.

**Figure 5:**
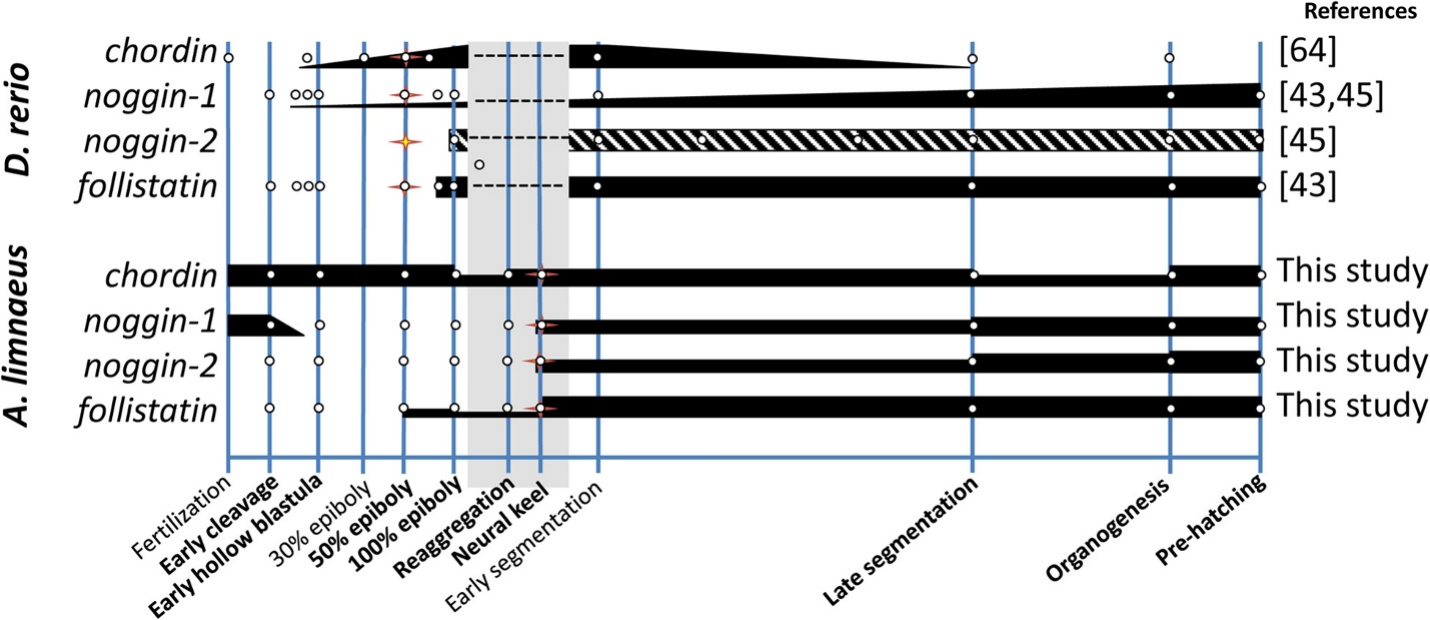
**Gene expression patterns of** ***chordin*** **,** ***noggin-1*** **,** ***noggin-2*** **, and** ***follistatin*** **during development in** ***A. limnaeus*** **and zebrafish.** Developmental stages are based on *A. limnaeus* timing and development. All data (white dots) are inferred from available qPCR or northern blot data, except for zebrafish *noggin-2* which was inferred from whole embryo *in situ* hybridization (noted by the striped bar). Thickness of the black bars represents relative expression levels within each species. The first visible evidence of embryogenesis is indicated with a star for both species. For data from this study, late segmentation and organogenesis stages would correspond to the DII and 9dpd stages, respectively. Developmental stages that are absent from zebrafish are indicated with a gray box.

Only one *chordin* gene has been identified in zebrafish and medaka and its expression is required for correct dorsal structure formation [44, 45, 66, 67]. Expression of *chordin* is first observed in the late blastoderm of both medaka and zebrafish, just prior to epiboly, and transcripts appear to be expressed until around the end of somitogenesis for both species. We detected *chordin* expression throughout *A. limnaeus* embryonic development (Figure 4A) with peak expression just after fertilization followed by a sharp drop after the completion of epiboly (4 dpf). This pattern suggests maternal packaging of the chordin transcript, which appears to be a unique expression pattern, compared to zebrafish (Figure 5), and may indicate a role for *chordin* expression in the unique dispersion and reaggregation phases of development observed in annual killifish.

#### noggin-1 and noggin-2

At least three *noggin* genes have been described in zebrafish, with expression of *noggin-1* starting at the late blastula stage and *noggin-2* appearing at the end of gastrulation [41]. High expression of *noggin-1* was observed just after fertilization in *A. limnaeus* (EC stage), after which expression became undetectable until the SNK stage at 10 dpf (Figure 4B). The constitutive expression of zebrafish *noggin-1*, beginning shortly before gastrulation and continuing for the duration of embryonic development (Figure 5), contrasts with this expression profile of *A. limnaeus noggin-1*[41, 43]. Expression of *noggin-2* was first detected at the SNK stage in *A. limnaeus* in association with the formation of the embryonic axis, similar to zebrafish where *noggin-2* is first detected in the axial mesoderm at the end of gastrulation [41]. Expression of *noggin-2* continued to increase during development reaching peak levels in mid to late organogenesis (3/4 OG) and DIII embryos (Figure 4C). After the SNK stage, expression of both *noggin-1* and *noggin-2* was observed for the remainder of the embryonic stages, suggesting roles in DV patterning and neural development similar to other teleosts during these periods of development [41, 43, 68].

#### follistatin

While localization studies in zebrafish have determined that chordin and noggin are likely secreted as part of the organizer, follistatin appears to be absent in this structure [43, 45]. *A. limnaeus follistatin* expression is first measurable at 50% EB and increases dramatically between the late reaggregation and SNK stages of development (8 to 10 dpf) after which it is expressed constitutively until the end of embryonic development (Figure 4D). This timing of *follistatin* expression in *A. limnaeus* is similar to zebrafish (Figure 5) in that high expression of transcripts is not seen until the embryonic axis is visible, suggesting a role outside of the early gastrulation processes [43].

Expression of *chordin*, *noggin-1*, and *noggin-2* at the first appearance of a visible embryonic axis, suggests that these factors may play a role in DV patterning in *A. limnaeus* that is similar to other described vertebrates, such as zebrafish (Figure 5). However, contrasting to zebrafish embryos, the surprising observation of maternally packaged *chordin* and *noggin-1* during early cleavage suggests that these genes may have a unique function during early development in *A. limnaeus*. Transcript localization and protein expression studies for these genes will be necessary to determine where these genes are expressed during development and may clarify their roles in *A. limnaeus* morphogenesis.

### The dispersed blastomere stage may prolong an undifferentiated state that can buffer environmental stress

In zebrafish, previously reported transcript data shows that simultaneous expression of *chordin*, *noggin-1*, *noggin-2*, *follistatin*, and *sox2* is only observed after commencement of germ layer formation. Similarly, we do not observe simultaneous expression of these five genes until after formation of the solid neural keel when there is a visible embryonic axis (Figures 3 and 5). Cross-species comparisons of *sox2* transcripts also reveal that *sox2* expression is generally strongest following gastrulation (Figure 3), similar to our observation of highest *sox2* expression following axis formation (Figure 2). Taken together, expression profiles of the genes tested in this study suggest that gastrulation and axis formation in *A. limnaeus* is not completed until late in the reaggregation process or perhaps shortly after (between 8 and 10 dpf).

Wourms [2] suggested that the dispersed cells might be ‘developmentally equivalent’ and therefore able to replace cells that are damaged or destroyed after environmental insult. Recently, we have shown that *A. limnaeus* embryos irradiated with ultraviolet-C (254 nm) during the dispersed blastomere phases (4 dpf) suffer a delay in development, but are able to develop normally at doses that cause a high degree of abnormal development in SNK stage embryos [21]. Taken together with the gene expression profiles in this report, these data support the hypothesis that the dispersed cell phase in annual killifish can serve to buffer development against potentially teratogenic insults through the prolonged maintenance of pluripotency and the delay of sensitive developmental processes (for example, gastrulation and axis formation) until the environment is favorable for normal development. The fact that many species of annual killifish can either substantially prolong D/R or enter diapause at this stage in response to environmental stress [3, 4, 11] suggests a mechanism for embryos to survive prolonged bouts of what would otherwise be lethal or teratogenic environmental stress. To our knowledge, this is the first evidence supporting this type of mechanism for dealing with environmental stress during development.

### Gene expression during diapause II and implications for the annual killifish life history

In all lineages of annual killifish, the processes of D/R and the ability to arrest development in diapause always co-occur despite the lack of a necessary connection between the two processes. This has led us to hypothesize that the molecular mechanisms that support the two processes may be somehow linked. We observed mRNA expression of *oct4*, *sox3*, *sox2*, *chordin*, *follistatin*, *noggin-1*, and *noggin-2* in DII embryos. Perhaps most interesting is the high and prolonged expression of *chordin* for the entire duration of embryonic development in *A. limnaeus* which is in contrast to the pattern observed in zebrafish (Figure 5). While the genes investigated in this study are of central importance to normal development, it is not clear why their expression would be maintained during diapause II. Previous work suggests low rates of protein turnover during diapause II, but the reduction is not complete and some transcripts are very likely translated during dormancy [69]. Presently, it is unclear if these seven mRNAs in DII embryos are: (1) actively translated and have a role in maintaining diapause; (2) stabilized in order to support rapid resumption of development once diapause is terminated; or (3) leftover from pre-DII development. Further studies of the action of these genes before, during, and after diapause II, especially the prolonged expression of *chordin*, might lead to interesting discoveries on the molecular regulation and evolution of this complex developmental pattern.

## Conclusions

The expression of *oct4*, *sox3*, *sox2*, *chordin*, *follistatin*, *noggin-1*, and *noggin-2* are dynamic across embryonic development in *A. limnaeus*. Our data suggest that *A. limnaeus* embryonic cells begin to acquire specific identities after the D/R process is completed and gastrulation commences. The dispersed blastomeres during the D/R stage are very likely pluripotent and lack spatial orientation, and this may benefit the embryos by delaying the sensitive process of gastrulation and axis formation until environmental conditions are favorable. The implications for this work reach far beyond the peculiar developmental patterns of annual killifish, and suggest that even highly conserved developmental pathways that are required for the formation of the basic vertebrate body plan can be altered in response to intense selective pressure to generate unique life histories and alter the timing of early development.

## Electronic supplementary material

Additional file 1: Table S1: Degenerate primers for fragment isolation. (DOCX 73 KB)

Additional file 2: Table S2: Isolated and cloned *A. limnaeus* gene fragments. (DOCX 125 KB)

Additional file 3: Table S3: Blast results of cloned fragments. (DOCX 51 KB)

Additional file 4: Table S4: qPCR primers and probes. (DOCX 69 KB)

Additional file 5: Table S5: Top blastx searches to *D. rerio.* (DOCX 36 KB)

## Abbreviations

ANOVA: Analysis of the variance
BMP: Bone morphogenetic protein
EtOH: Ethanol
dpf: Days post fertilization
dpd: Days post diapause
DI: Diapause I
DII: Diapause II
DIII: Diapause III
D/R: Dispersion-reaggregation
DV: Dorsal-ventral
FAM: Fluorescein
PCR: Polymerase chain reaction
qPCR: Quantitative polymerase chain-reaction
SMO: Spemann-Mangold organizer
EC: Early cleavage
EHB: Early hollow blastula
50% EP: 50% epiboly
DBP: Dispersed blastomere phases
RP: Reaggregation phases
SNK: Solid neural keel
3/4 OG: Three-quarters overgrowth.
