# Supplementary material for: Gene expression patterns that support novel developmental stress buffering in embryos of the annual killifish Austrofundulus limnaeus
Source: EvoDevo. 2015 Jan 21;6:2. doi: 10.1186/2041-9139-6-2 (PMC4372997; doi:10.1186/2041-9139-6-2)
Supplement: Supplementary file 1 — Additional file 1: Table S1: Degenerate primers for fragment isolation. (DOCX 73 KB) [file 13227_2014_139_MOESM1_ESM.docx]

| **Table S1: Degenerate primers for fragment isolation** | | | | |
| --- | --- | --- | --- | --- |
| **Gene** | **F primer** | **R primer** | **Species used with accession IDs** | **PCR cycling** |
| *oct4* | GAGGCTCTSCARCTKAGYTTC | RGATGTRGTVCGWGTDTGGTT | *Danio rerio* [GenBank:NM_131112.1], *Homo sapiens* [GenBank:NM_002701.4], *Labeo rohita* [GenBank:GU443948.1], *Pagrus major* [GenBank:AB378582.1] | Initial: 30 sec, 94ºC  37 cycles: 30 sec, 50ºC > 30 sec, 72ºC  End: 7 min, 72ºC |
| *sox2* | CAAGACCCTCATGAAGAAGGAC | TBCAGTACAACTCCATGACYA | F primer: *Oreochromis niloticus* [ GenBank:EF431920.1], *Takifugu rubripes* [GenBank:AY277952.1], *Epinephelus coioides* [GenBank:FJ432695.1]  R primer: *Danio rerio* [GenBank:NM_213118.1], *Oryzias latipes* [GenBank:FJ895588.1], *Salmo salar* [GenBank:NM_001141718.1] | Initial: 30 sec, 95ºC  5 cycles: 20 sec, 95ºC > 20 sec, 63ºC > 1.5 min, 68ºC  5 cycles: 20 sec, 95ºC > 20 sec, 58ºC > 1.5 min, 68ºC  35 cycles: 20 sec 95ºC > 20 sec, 53ºC > 1.5 min, 68ºC  End: 7 min, 72ºC |
| *sox3* | AACCCMAAAATGCACAAYTC | ATGAACGGCTGGACMAAY | *Danio rerio* [GenBank:NM_213118.1], *Oryzias latipes* [GenBank:FJ895588.1], *Salmo salar* [GenBank:NM_001141718.1] | Initial: 30 sec, 95ºC  5 cycles: 20 sec, 95ºC > 20 sec, 65ºC > 1.5 min, 68ºC  5 cycles: 20 sec, 95ºC > 20 sec, 60ºC > 1.5 min, 68ºC  36 cycles: 20 sec, 95ºC > 20 sec, 55ºC > 1.5 min, 68ºC |
| *chordin* | AGCRGGTCAYGCMTGGGT | CACTGYGAGAAGGTGACST | *Danio rerio* [GenBank:NM_130973.1], *Xenopus laevis* [GenBank:NM_001088309.1] | Initial: 30 sec, 95ºC  5 cycles: 20 sec, 95ºC > 20 sec, 65ºC > 1.5 min, 68ºC  5 cycles: 20 sec, 95ºC > 20 sec, 60ºC > 1.5 min, 68ºC  30 cycles: 20 sec, 95ºC > 20 sec, 55ºC > 1.5 min, 68ºC |
| *noggin-1* | ANCTNAACGAGACCGAGCT | TCACAGASTGCAAATGCTCM | *Danio rerio* [GenBank:AF159147.1], *Fugu ribripes* [GenBank:AF095337.1] | Initial 30 sec, 95ºC  5 cycles: 20 sec, 95ºC > 20 sec, 65ºC > 1.5 min, 68ºC  5 cycles: 20 sec, 95ºC > 20 sec, 60ºC > 1.5 min, 68ºC  36 cycles: 20 sec, 95ºC > 20 sec, 55ºC > 1.5 min, 68ºC  End: 7 min, 72ºC |
| *noggin-2* | CTGTGGNCCTACTCNTTCTGC | TCACAGASTGCAAATGCTCM | *Danio rerio* [GenBank:AF159147.1], *Fugu ribripes* [GenBank:AF095337.1] | Same as *noggin-1* |
| *follistatin* | CTCATGGAACATCAAAAAGTTCAA | CGACAACACCACATATCCCA | *Oreochromis mossambicus* [GenBank:DQ343148.1], *Micropterus salmoides* [GenBank:EF128004.1], *Larimichthys crocea* [GenBank:JF499691.1], *Takifugu rubripes* [GenBank:NM_001037858.1], *Sparus aurata* [GenBank:AY544167.1] | Initial: 30 sec, 95ºC  5 cycles: 20 sec, 95ºC > 20 sec, 65ºC > 1.5 min, 68ºC  5 cycles: 20 sec, 95ºC > 20 sec, 60ºC > 1.5 min, 68ºC  30 cycles: 20 sec, 95ºC > 20 sec, 55ºC > 1.5 min, 68ºC  End: 7 min, 68ºC |
