# Supplementary material for: Gene expression patterns that support novel developmental stress buffering in embryos of the annual killifish Austrofundulus limnaeus
Source: EvoDevo. 2015 Jan 21;6:2. doi: 10.1186/2041-9139-6-2 (PMC4372997; doi:10.1186/2041-9139-6-2)
Supplement: Supplementary file 2 — Additional file 2: Table S2: Isolated and cloned A. limnaeus gene fragments. (DOCX 125 KB) [file 13227_2014_139_MOESM2_ESM.docx]

| **Table S2. Isolated and cloned *A. limnaeus* gene fragments.** | | | |
| --- | --- | --- | --- |
| **Putative *A. limnaeus* gene** | **Stage(s) used^a^** | **Cloned fragment^b^** | **Size (bp)** |
| *oct4* | 3 dpf | GAGGCTCTGCAGCTGAGCTTCAAGAACATGTGCAAGCTGAAGCCCCTTCTGCAGAGATGGCTGGTCGAGGCAGAGACCTCGGAAAATCCTCA**AGATATGTACAAGGTTGAGCGAGTTTTTGTCGACACCAGAAAAAGGAAGAGGAGGACCAGCCTGGAGGGAGCGGTGCGCTCCGCCCTGGAGTCCTTCTACATCAAGTGTCCCAAACCGAACACTCAGGAAATCACA**CAAATCGCAGACGAGTTGGGCCTGGAAAGAGATGTGGTGCGTGTTTGGTT | 278 |
| *sox2* | 4 dpd – 8 dpd | TAGTCATGGAGTTGTACTGGAGCGCGCTCATGTCGTAGCGGTGCATGGGCTGCATCTGGCCCGCGCCGTGCGCGTTCAGGCCCGGGTGCTGG**TAGCTCAGCTGCTCCTGCATCATCCCGTAGCCGCCGTTGGTCCAGCCGTTCATGTGCGCCGCGTAGCTGTCCATCCTCTGGTTCA**CCCCGCCGCCCAGCCCGGCCCCGACCCCGACCCCGGTGCCCATCCCGTTGCCCCCGGGCGCCAGCAGCCCCCCCGGCAGGGTGTACTTGTCCTTCTTCATGAGGGTCTTG | 287 |
| *sox3* | 4 dpd – 8 dpd | AACCCCAAAATGCACAACTCTGAAATCAGCAAGCGGCTCGGCGCC**GACTGGGAAACTTCTGACGGACGCGGAGAAGAGGCCGTTCATCGACGAGGCCAAGCGGCTGCGCGCGATGCACATGAAAGAGCACCCGGATTATAAATACCGGCCCCGCAGGAAGACCAAGACCCTGCTCAAGAAA**GACAAGTATTCTTTGCCCGGGGGTCTGTTGGCGCCAGGATCCAACGCCGTCAACAACTCGGTGTCGGTGGGGCAGCGCATGGACGGTTACGCGCACATGAACGGCTGGACCAAC | 295 |
| *chordin* | 10 dpf | GCGTGGGTGTCAGTGGACAAACAGTGCCACCTGCACTATGAAATAGTAGTCGCGGGTCTGAGTAAGAAGGAGGACGTCACTGTGAATGCCCACCTACATGGACTGGCTGAGATCGGGGAGCTGGACAACAGCAGCACCACACACAAGAGGCTGCTGACGGGCTTCTATGGCTCCCAGGCTCAGGGGATTTTAAAGGACCTTAGTCTTGAATTACTGCAACACCTGGACCAGGGAACGGCCTTCATCCAAGTCAGCACCAAGCTGAATCCCCAAGGAGAAATACGAGGACGGGTCCATGTTCCAAACAGCTGCGAGTCTGGAGCCGGAGCCGAGGTGGAGGAGGCCGAGTTGGATGACACGTTGCTGACAGATCCCGAGGAACTAAAGAAAGACCCCAACACTTGTTTTTTTGAGAACCAGCACCACGCTCATGGTTCCCGCTGGACGCCTAACTATGACAAGTGTTTCACCTGCAGCTGCCAGAAGAGGACAGTAATCTGTGATCC**TGTCATCTGTCCCGTGTTGACCTGCTCCAGAACCATTCAGCCTGAGGACAAGTGCTGTCCCATCTGTGATGAAAAGCGAGAGCCCAAAGACATGAA**AGCTGGAGAGAAGGTGGAAGAACATCCTGAAGGGTGTTACTCTGAAGGAGATCAGAAAATGCATGCTCCAGGAATATCATGGCATCCGTTTGTTCCTCCATTTGGCTACATTAAATGTGCTGTCTGCACCTGCAAGGGGTCTTCAGGGGAGGTCCACTGCGAGAAGGTGAC | 773 |
| *noggin-1* | 8 dpd – 9 dpd | CTGAACGAGACCGAGCTGAGGAGCATCCTGGGGGAGTTTGACCTGCGCTTTTTGTCCGCGTCCGTCCCCGCGGAGGACAGGTTCCCGGGGAACGACGAGCTGGACGCGTCCGACAGCCTGAGGGCGGGCGGCGGGATGATGCCCAAAGAGATCCGCGCCGTGGACTTCGACGTCCAGGTGGG**CAAGAAACAGAAACCCAGCAAGAAGCTGAAGCGGCGGCTGCAGCAGTGGCTGTGGGCCTACACCTCCTACCCGGTTCTGTACA**GCTGGACCGACCTGGGGATCCGCTTCTGGCCGCGCCACGTGCGCGCGGCCAGCTGTCTGAGCAAGAGGTCGTGCTCCGTCCCGGAAGGGATGGTCTGCAAACCCGCGAACTCGACCCACCTGACGCTGCTGAGGTGGAGGTGCGTGCAGAGGAAGGCGGGGCTGAAGTGCGCGTGGATCCCGATCCAGTACCCGCTCATCACAGACTGCAAATGCTCC | 483 |
| *noggin-2* | 8 dpd – 9 dpd | CTGTGGACCTACTCTTTCTGCCCTGTGGTGTACACCTGGAAGGACCTGGGCGTGAGGTTCTGGCCGCGCTACATCAAGGAGGGCAACTGTTTCTCCGAGCGCTCGTGCTCCTTCCCGGAGGGGATGTCCTGCAAGCCC**GTCAAGTCGGTCACCAAGATCTTCCTGCGGTGGTACTGCCAGGGCTTTCTAAGACAGAAATACTGTACGTGGATACAGGTGCAAT**ACCCGGTCATCACAGACTGCAA | 245 |
| *follistatin* | 8 dpd – 9 dpd | CACCCTCTTTAGGTGGATGATCTTCAATGGGGGCGCGCCTAATTGCATACCTTGCAAAGGTG**GAGAAAGCTGCGAAAACGTGGACTGTGGGCCCGGGAAGACGTGCAAGATCAACCGCAAGGGGAAGCCGCGCTGCGTGTGCGCGCCCGACTGCTCCAACATCACCTGGAAGGGACCCGTCTGCGGCACAGACGGCAAGACCTACAAA**AACGAGCGCGCGCTCCTGAGGGCGAAATGCAAAACCAACCCGGACCTGGACGTGCAGTACCAGGGCAAATGCAAGAAAACGTGCCGTCAGGTCTTGTGCCCCGGGACCTCCAACTGCGTTGTGGACCAGACGAACAACGCGTACTGCGTGACGTGTAATCGGATTTGCCCCCCCGCGACCTCGCCTGATCTGTACCTGTGTGGAAACGACGGGATCACATATCCGAGCGCGTGCCACCTGAGAAAGGCGACCTGTCTGCTCGGCAGGTCCATCGGGGTGGCGTACGACGGAAAATGCATCAAGGCCAAGTCGTGTCAGGCCATCACGTGCAGCGCAGGAAAGAAGTGTCTGTGGGACGATCGGACGGGCCGGGGCCGCTGCTCGGTCTGCGTGGACCCCTGTCCACAGAGCCGGCCGAACGAGGCGGTGTGTGCCAGCGACAACACCACATATCCCA | 665 |
| *β-actin* | 3 dpf | **CTGGAACGATGAAGGAAACTTAATGTTTTGGCTATGTTTAATAAAAACACCCGGTTCATCTGGGGACTTAAAAATGTACCTTTTTGTCATTCCAAATGTTTGTTAACTGCATTGTTCAGACATGATTCC**AGATGTTAACTGCATTGTTCAGACACGTATTTGCCTCTGTGAAGGCTGCCCAGTGGTTGGCGCATACTTTAACATGGTTGTAGTATCGCTTGTATGTAAATTTGTCTGGGGTTTTTTGTACTTTCAGCCTTAAAGAAAAACTTGGTCCTGTTTAA | 284 |
| 18s rRNA | 3 dpf | GAGGCTCTGCAGCTGAGCTTCCGGCCGGGGCCCGAAGCGTTTACTTTGAAAAAATTAGAGTGTTCAAAGCAGGCCCGGTCGCCTGAATACCGCAGCCAGGAATAATGGAATAGG**ACTCCGGTTCTATTTTGTGGGTTTTCCCTGAACTGGGGCCATGATTAAGAGGGACGGCCGGGGGCATTCGTATTGTGCCGCTAGAGGTGAAATTCTTGGA**CCGGCGCAAGACGGACGAAAGCGAAAGCATTTGCCAAGAATGTTTTCATTAATCAAGAACGAAAGTCGGAGGTTCGAAGACGATCAGATACCGTCGTAGTTCCGACCATAAACGATGCCGACTAGCGATCCGGCGGCGTTATTCCCATGACCCGCCGGGCAGCGTCCGGGAAACCAAAGTCTTTGGGTTCCGGGGCGAGTATGGTTCTGCAAC | 427 |
| a. dpf, days post-fertilization; dpd, days post-diapause.  b. The amplicon sequence used for qPCR analysis is noted in bold for each fragment. | | | |
