# Supplementary material for: Gene expression patterns that support novel developmental stress buffering in embryos of the annual killifish Austrofundulus limnaeus
Source: EvoDevo. 2015 Jan 21;6:2. doi: 10.1186/2041-9139-6-2 (PMC4372997; doi:10.1186/2041-9139-6-2)
Supplement: Supplementary file 3 — Additional file 3: Table S3: Blast results of cloned fragments. (DOCX 51 KB) [file 13227_2014_139_MOESM3_ESM.docx]

| **Table S3: Blast results of cloned fragments** | | | | | | |
| --- | --- | --- | --- | --- | --- | --- |
| **Putative *A. limnaeus* gene** | **Top blast hit** | **Algorithm** | **Identity** | **E value** | **Query Coverage** | **GenBank Accession** |
| *oct4* | PREDICTED: POU domain, class 5, transcription factor 1-like [*Stegastes partitus*] | blastx | 92% | 9e-53 | 99% | XP_008302560.1 |
| *sox2* | PREDICTED: transcription factor SOX-2 [*Cynoglossus semilaevis*] | blastx | 95% | 2e-30 | 99% | XP_008336454.1 |
| *sox3* | PREDICTED: transcription factor Sox-3-like [*Xiphophorus maculatus*] | blastx | 100% | 1e-32 | 100% | XP_005799559.1 |
| *chordin* | PREDICTED: chordin-like [*Poecilia formosa*] | blastx | 88% | 7e-148 | 99% | XP_007562667.1 |
| *noggin-1* | PREDICTED: noggin-like [*Maylandia zebra*] | blastx | 78% | 2e-75 | 100% | XP_004556443.1 |
| *noggin-2* | noggin2 [*Trachinotus blochii*] | blastx | 91% | 2e-49 | 99% | ACX71862.1 |
| *follistatin* | PREDICTED: follistatin-A-like isoform X2 [*Oreochromis niloticus*] | blastx | 84% | 3e-132 | 99% | XP_005473610.1 |
| *β-actin* | *Scatophagus argus* beta-actin mRNA | blastn | 87% | 7e-82 | 98% | KF649214 |
| 18S rRNA | *Nothobranchius furzeri* 18S rRNA | blastn | 97% | 0.0 | 97% | N603833.1 |
