# Supplementary material for: Gene expression patterns that support novel developmental stress buffering in embryos of the annual killifish Austrofundulus limnaeus
Source: EvoDevo. 2015 Jan 21;6:2. doi: 10.1186/2041-9139-6-2 (PMC4372997; doi:10.1186/2041-9139-6-2)
Supplement: Supplementary file 4 — Additional file 4: Table S4: qPCR primers and probes. (DOCX 69 KB) [file 13227_2014_139_MOESM4_ESM.docx]

| **Table S4: qPCR primers and probes** | | | | |
| --- | --- | --- | --- | --- |
| **Putative *A. limnaeus gene*** | **F primer** | **R primer** | **Probe^a^** | **Amplicon size (bp)** |
| *oct4* | AGATATGTACAAGGTTGAGCGAG | TGTGATTTCCTGAGTGTTCGG | /6-FAM/TTGATGTAG/ZEN/AAGGACTCCAGGGCG/IBFQ/ | 136 |
| *sox2* | TAGCTCAGCTGCTCCTGCATCAT | TGAACCAGAGGATGGACAGCTAC | /6-FAM/CGTTGGTCC/ZEN/AGCCGTTCATGTG/IBFQ/ | 85 |
| *sox3* | GACTGGGAAACTTCTGACGG | TTTCTTGAGCAGGGTCTTGG | /6-FAM/TGCACATGA/ZEN/AAGAGCACCCGGAT/IBFQ/ | 136 |
| *chordin* | TTCATGTCTTTGGGCTCTCG | TGTCATCTGTCCCGTGTTG | /6-FAM/TTGTCCTCA/ZEN/GGCTGAATGGTTCTGG/IBFQ/ | 96 |
| *noggin-1* | TGTACAGAACCGGGTAGGAG | CAAGAAACAGAAACCCAGCAAG | /6-FAM/AGCAGTGGC/ZEN/TGTGGGCCTA/IBFQ/ | 83 |
| *noggin-2* | GTCAAGTCGGTCACCAAGATC | ATTGCACCTGTATCCACGTAC | /6-FAM/CTGTCTTAG/ZEN/AAAGCCCTGGCAGTACC/IBFQ/ | 85 |
| *follistatin* | GAGAAAGCTGCGAAAACGTG | TTTGTAGGTCTTGCCGTCTG | /6-FAM/CTTCCCCTT/ZEN/GCGGTTGATCTTGC/IBFQ/ | 146 |
| *β-actin* | TGGAACGATGAAGGAAACTTAATG | GGAATCATGTCTGAACAATGCAG | /6-FAM/TCCCCAGAT/ZEN/GAACCGGGTGTTTT/IBFQ/ | 129 |
| 18S rRNA | ACTCCGGTTCTATTTTGTGGGT | TCCAAGAATTTCACCTCTAGCG | /6-FAM/CCGTCCCTC/ZEN/TTAATCATGGCCCC/IBFQ/ | 100 |

a. Placement of dye and quenchers are indicated in the probe sequence: 6-FAM, 5’ labeled fluorescein; ZEN, internal ZEN fluorescence quencher; IBFQ, 3’ Iowa Black fluorescence quencher.
