# Supplementary material for: Gene expression patterns that support novel developmental stress buffering in embryos of the annual killifish Austrofundulus limnaeus
Source: EvoDevo. 2015 Jan 21;6:2. doi: 10.1186/2041-9139-6-2 (PMC4372997; doi:10.1186/2041-9139-6-2)
Supplement: Supplementary file 5 — Additional file 5: Table S5: Top blastx searches to D. rerio. (DOCX 36 KB) [file 13227_2014_139_MOESM5_ESM.docx]

| **Table S5: Top blastx searches to *D. rerio*** | | | | | |
| --- | --- | --- | --- | --- | --- |
| **Putative *A. limnaeus gene*** | ***Danio rerio* top blastx hit** | **Identity** | **E value** | **Query Coverage** | **GenBank Accession** |
| *oct4* | OCT4/POU5f1 | 88% | 7e-52 | 99% | Q90270.1 |
| *sox2* | SOX2 | 91% | 2e-26 | 91% | NP_998283.1 |
| *sox3* | SOX3 | 95% | 2e-29 | 100% | NP_001001811.2 |
| *chordin* | Chordin | 70% | 1e-122 | 99% | AAI62594.1 |
| *noggin-1* | Noggin-1 | 68% | 4e-63 | 100% | NP_571058.2 |
| *noggin-2* | Noggin-2 precursor | 86% | 3e-47 | 99% | NP_571067.1 |
| *follistatin* | Follistatin | 81% | 7e-126 | 99% | AAH78241.1 |
